# Risk of Esophageal Cancer Following Percutaneous Endoscopic Gastrostomy in Head and Neck Cancer Patients: A Nationwide Population-Based Cohort Study in Taiwan

**DOI:** 10.1097/MD.0000000000002958

**Published:** 2016-03-07

**Authors:** Kuen-Tze Lin, Chun-Shu Lin, Shih-Yu Lee, Wen-Yen Huang, Wei-Kuo Chang

**Affiliations:** From the Department of Radiation Oncology (K-TL, C-SL, W-YH), Tri-Service General Hospital; Graduate Institute of Aerospace and Undersea Medicine (S-YL); and Division of Gastroenterology (W-KC), Department of Internal Medicine, Tri-Service General Hospital, National Defense Medical Center, Taipei, Taiwan.

## Abstract

Esophageal cancers account for majority of synchronous or metachronous head and neck cancers. This study examined the risk of esophageal cancer following percutaneous endoscopic gastrostomy (PEG) in head and neck cancer patients using the Taiwan National Health Insurance Research Database.

From 1997 to 2010, we identified and analyzed 1851 PEG patients and 3702 sex-, age-, and index date-matched controls.

After adjusting for esophagitis, esophagus stricture, esophageal reflux, and primary sites, the PEG cohort had a higher adjusted hazard ratio (2.31, 95% confidence interval [CI] = 1.09–4.09) of developing esophageal cancer than the controls. Primary tumors in the oropharynx, hypopharynx, and larynx were associated with higher incidence of esophageal cancer. The adjusted hazard ratios were 1.49 (95% CI = 1.01–1.88), 3.99 (95% CI = 2.76–4.98), and 1.98 (95% CI = 1.11–2.76), respectively.

Head and neck cancer patients treated with PEG were associated with a higher risk of developing esophageal cancer, which could be fixed by surgically placed tubes.

## INTRODUCTION

Most advanced-stage head and neck cancer patients receive concurrent chemoradiotherapy, which commonly leads to severe dysphagia, odynophagia, and dehydration. In such patients, percutaneous endoscopic gastrostomy (PEG) is commonly used (in approximately 46%) for enteral nutritional support.^1–3^ Many studies have pointed out that a rare but severe complication of PEG in primary head and neck cancer patients is metastasis to the stomach stoma site (metastasis rate, approximately 1.3%).^4–13^ In theory, when the PEG tube is placed from the upper digestive tract to a stoma in the abdominal wall, the tube may contact the esophagus and consequently lead to esophageal metastasis. However, there has been no previous report of subsequent esophageal cancer in a head and neck cancer patient after PEG.

Esophageal and lung cancers account for the majority of synchronous or metachronous head and neck cancers. The frequency of a second malignancy in head and neck cancer patients is relatively high, and the survival following a second cancer is poor; the 5-year survivals in head and neck patients who develop second malignancies in the esophagus and lung are only 2.6% and 2.4%, respectively.^14^ Accordingly, to identify and decrease the risk of a second primary cancer of the esophagus is important, because it implies poor survival and disease control.^14–22^

In 1996, Taiwan started its National Health Insurance (NHI) program, a single-payer and universal insurance plan, with a coverage rate of 97% among the hospitals and clinics. In 1998, a total of 99% of the people in Taiwan received health care given by the NHI.^23^ The NHI has created the Taiwan National Health Insurance Research Database (NHIRD) for researchers in Taiwan, which has been widely applied in epidemiologic and clinical studies.^24–27^ The NHIRD contains the annual registration files and original claims data for reimbursement, and is managed by the National Health Research Institutes.

In this study, we performed a large-scaled nationwide retrospective cohort study using the NHIRD to investigate the risk of esophageal cancer following PEG in head and neck cancer patients.

## METHODS

### Data Source

We used the data from the NHIRD given by the Bureau of National Health Insurance of the Department of Health in Taiwan from the period of 1997 to 2010. In this study, we collected disease histories from inpatient files. The disease diagnoses were based on the International Classification of Diseases, Ninth Revision, Clinical Modification (ICD-9-CM). To protect patient privacy, all personal identification information is encrypted and deidentified before the data are released for research.

### Study Population

The present study was approved by the Institutional Review Board of the Tri-Service General Hospital, National Defense Medical Center (approval number 2-104-05-03), and the study protocol was performed in accordance with the Helsinki Declaration of 1975, as revised in 1983.

**FIGURE 1:**
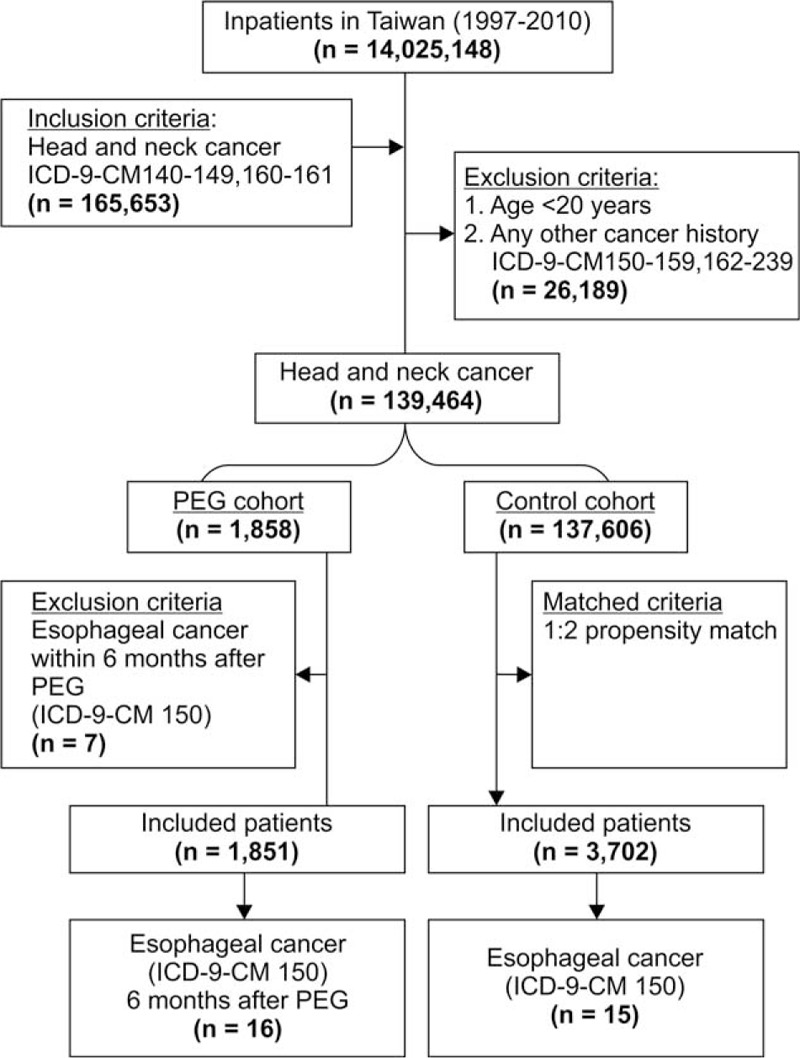
Flowchart of the study sample selection from the National Health Insurance Research Database. ICD-9-CM = International Classification of Diseases, Ninth Revision, Clinical Modification; PEG = percutaneous endoscopic gastrostomy.

We conducted a population-based retrospective cohort study (Figure 1) to clarify the relationship between PEG in head and neck cancer patients and the risk of developing esophageal cancer. We enrolled inpatients with newly diagnosed head and neck cancer (ICD-9-CM140-149,160-161) in Taiwan from 1997 to 2010. This study excluded patients with any other cancer (ICD-9-CM150-159,162-239) before the index date and patients aged <20 years. The index date was set as the PEG insertion date. A total of 139,464 patients were included. Head and neck cancer patients treated with PEG (n = 1858) were identified and classified as the PEG (ICD-9-CM OP 43.11) cohort. Patients with esophageal cancer (ICD-9-CM150) within 6 months before or after PEG were excluded (n = 7), resulting in a total of 1851 patients being included in the final PEG cohort. The control cohort consisted of patients without PEG (n = 137,606). We used 1:2 propensity score matching, considering the sex, age, and year of the index date, resulting in a total of 3702 patients being included in the final control cohort. The follow-up period was terminated upon developing esophageal cancer (ICD-9-CM 150), withdrawal from the insurance program, or by December 31, 2010 (Figure 1).

Based on the 2009 cancer registry annual report released by the Taiwan Department of Health,^28^ esophageal cancer is more frequent in males, with a median age at diagnosis of 57 years (67 years in females). Besides, patients with esophagitis, esophagus stricture, and esophageal reflux may display similar symptoms as patients with esophageal cancer. Furthermore, we postulated that different primary locations of the head and neck cancers may display different rates of metastasis to the esophagus. Accordingly, as all of the above may influence our results, we considered sex, age, esophageal cancer-associated comorbidities, and the primary tumor site of the head and neck cancer as confounding factors. Esophageal cancer-associated comorbidities included esophagitis (ICD-9-CM 530.1), esophagus stricture (ICD-9-CM 530.3), and esophageal reflux (ICD-9-CM 530.81). The primary tumor sites of the head and neck included the lips (ICD-9-CM 140), oral cavity (ICD-9-CM 141, 143-145), major salivary glands (ICD-9-CM 142), oropharynx (ICD-9-CM 146), nasopharynx (ICD-9-CM 147), hypopharynx (ICD-9-CM 148), nasal cavity and sinuses (ICD-9-CM 160), larynx (ICD-9-CM 161), and others (ICD-9-CM 149).

### Statistical Analysis

To demonstrate the differences between the PEG and control cohorts, the mean and standard deviation for continuous variables (age), and the count and percentage for category variables (sex, comorbidities, primary tumor sites) are presented. The *t* test for continuous variables and the *χ*^2^ test or Fisher exact test for categorical variables were used to statistically examine the differences between the 2 cohorts. The cumulative esophageal cancer incidence and demographic-specific and comorbidity-specific esophageal cancer incidence for the PEG and control cohorts were compared using a Cox's Proportional Hazards regression model adjusted for potential confounding factors to estimate the hazard ratios (HRs) and 95% confidence intervals (CIs) for the PEG cohort.

We used SAS 9.3 software (SAS Institute, Cary, NC) to manage and analyze the data. The significance level was set at <0.05, and all tests were 2-sided.

## RESULTS

### Patient Demographics and Follow-Up Period

A total of 1851 and 3702 patients were included in the PEG patient and control cohorts, respectively, with an identical mean age (54.7 years) and sex ratio (88.3% male; Table 1). The proportion of comorbidities in the PEG cohort was higher than that in the control cohort. There were a total of 46 patients with comorbidities (esophagitis, esophagus stricture, esophageal reflux) in the PEG cohort (2.5%), and 43 patients in the control cohort (1.2%) (*P* < 0.001). There were no significant differences in the proportions of the primary sites of the head and neck cancers between the 2 cohorts (*P* *=* 0.95, 0.93, 0.92, 0.89, 0.80, 0.81, 0.99, and 0.88 for the lips, oral cavity, major salivary glands, oropharynx, nasopharynx, hypopharynx, nasal cavities and sinuses, and larynx, respectively).

**TABLE 1:**
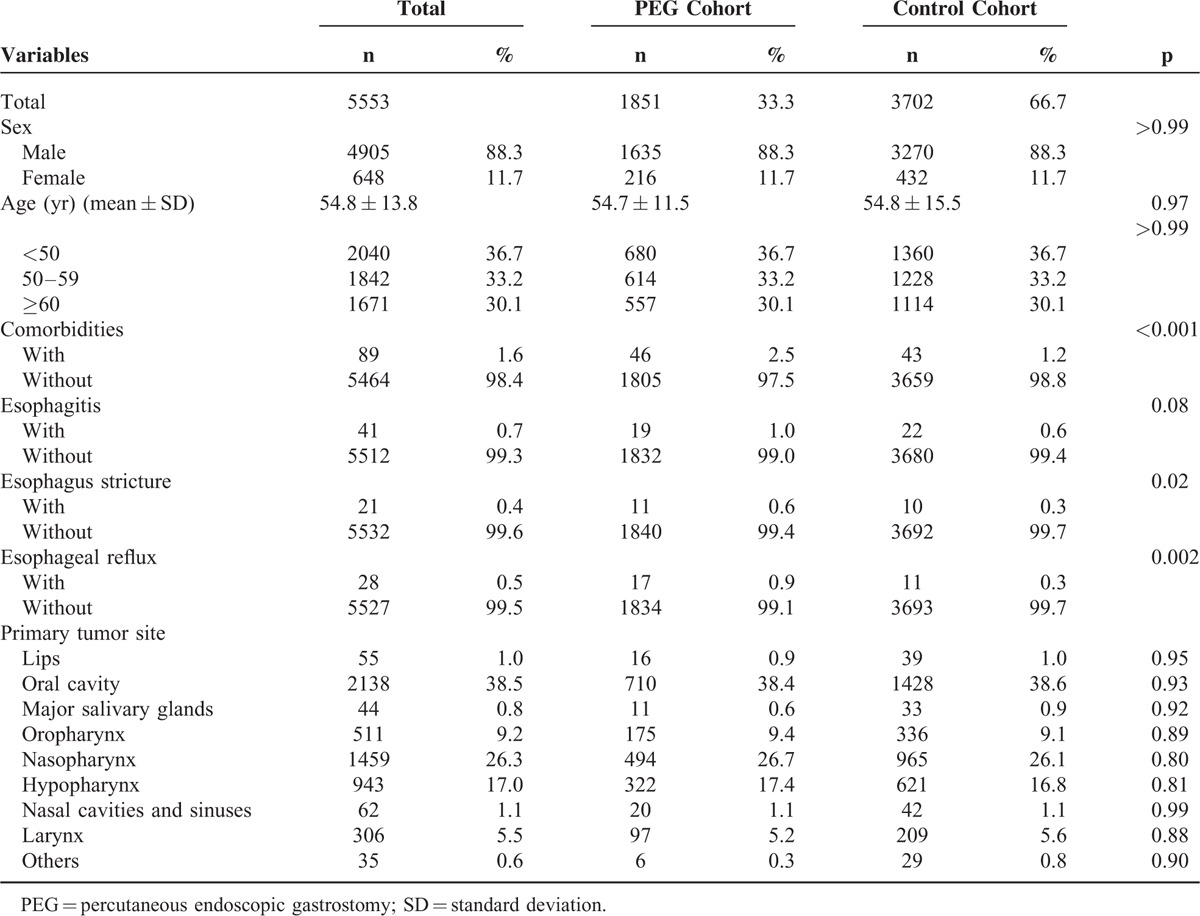
Baseline Demographics and Comorbidities in the 2 Study Cohorts

The average period for follow-up in the PEG cohort was 1.13 ± 1.69 years. Also, in this group, the maximum follow-up period was 10.77 years and minimum follow-up time was 0.01 years. In the cohort control group, the average follow-up time was 2.96 ± 3.13 years. In this group, the maximum follow-up period was 13.91 years and minimum follow-up period was 0.01 years.

### Risk Estimation

The incidence of esophageal cancer in the PEG cohort was 118.0 per 10,000 person-years. In the control cohort, the incidence was 51.2 per 10,000 person-years (Table 2). After adjusting for the possible confounding factors, the incidence of esophageal cancer in the PEG cohort was nearly 2.31-fold higher than that in the control cohort (HR = 2.31, 95% CI = 1.09–4.09).

**TABLE 2:**
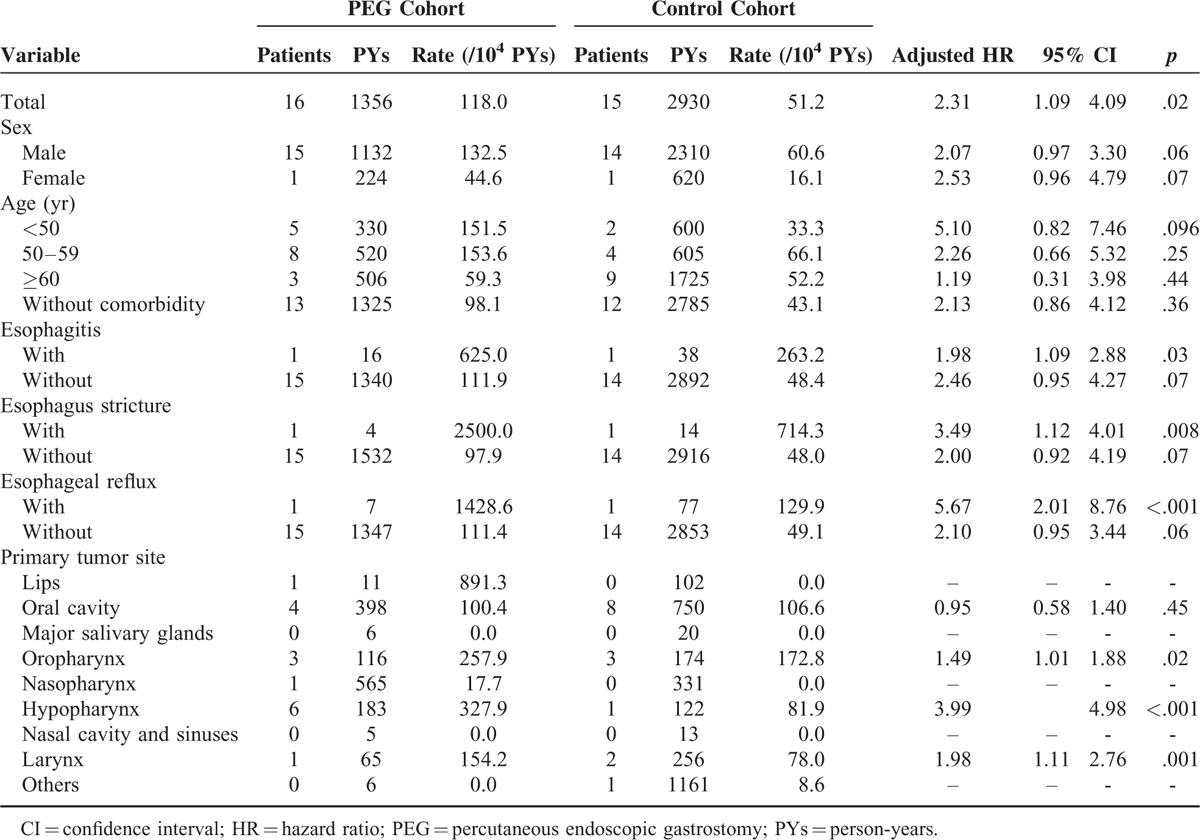
Incidence of Subsequent Esophageal Cancer and Multivariate Cox Proportional Hazards Regression Analysis of the Hazard Ratios in the 2 Study Cohorts

Table 2 shows the comorbidity-specific esophageal cancer incidence and the estimated HRs for both study cohorts. In the study population without any comorbidity, the PEG cohort still had a 2.13-fold higher risk of developing esophageal cancer than the control cohort (adjusted HR = 2.13, 95% CI = 0.86–4.12). Patients with esophagitis, stricture and stenosis of the esophagus, and esophageal reflux showed higher risks of developing esophageal cancer, with adjusted HRs of 1.98 (95% CI = 1.09–2.88), 3.49 (95% CI = 1.12–4.01), and 5.67 (95% CI = 2.01–8.76), respectively.

### Primary Tumor Locations in the Study Cohorts

Primary head and neck tumors located in the oropharynx, hypopharynx, and larynx were associated with a higher incidence of esophageal cancer (adjusted HRs, 1.49 [95% CI = 1.01–1.88], 3.99 [95% CI = 2.76–4.98]. and 1.98 [95% CI = 1.11–2.76], respectively; Table 2).

### Sensitivity Analysis

Lastly, we also used sensitivity analyses to assess the associations between PEG insertion and the risk of developing esophageal cancer according to different follow-up durations (Table 3). These findings suggested that, compared with the control cohort, the PEG cohort was associated with a significantly higher risk of developing esophageal cancer as the follow-up duration was increased. Especially, patients treated with PEG had a significantly greater incidence of developing esophageal cancer when the follow-up period was longer than 3 years; the adjusted HRs were 2.44 (95% CI = 1.00–4.86) for follow-up periods of 2 to 3 years and 5.24 (95% CI = 2.06–8.15) for follow-up periods of >3 years.

**TABLE 3:**
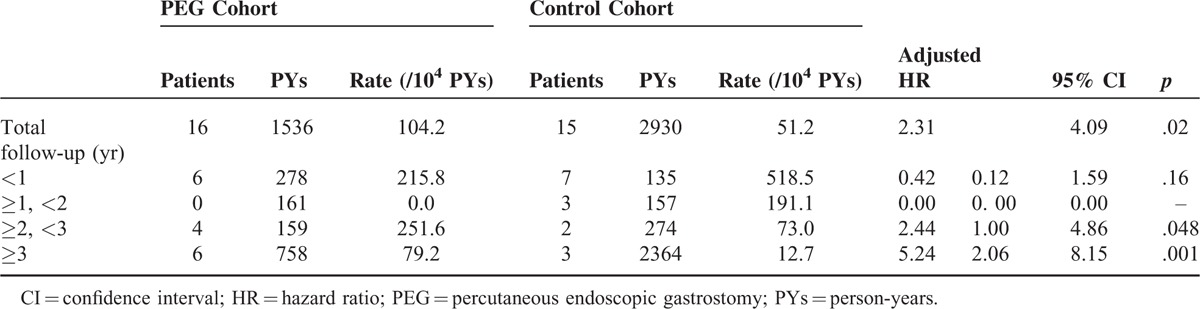
Cox Proportional Hazards Model for the Risk of Developing Esophageal Cancer in the Study Cohorts by the Follow-Up Year

## DISCUSSION

This is the first large-scale cohort study focusing on the correlation between the development of esophageal cancer and PEG insertion in head and neck cancer patients. In our findings, PEG insertion showed an association with the risk of subsequent esophageal cancer. After controlling for other important covariates, a 2.31-fold increased risk of developing esophageal cancer in head and neck cancer patients treated with PEG was noted.

To our knowledge, esophageal cancer is the most common metachronous and synchronous cancer in head and neck cancers, especially in oropharyngeal and hypopharyngeal cancer patients. The distribution of primary sites of head and neck cancers in both cohorts in this study was almost the same (Table 1), and to rule out the possibility of screening effects, we demonstrated the esophageal cancer incidence by follow-up years, which revealed that the risk was much higher when the time lag was longer than 3 years (Table 3).

Cancer metastasis at the PEG tube exit site in head and neck cancer patients is a rare but important problem. Possible mechanisms of this phenomenon include tumor seeding^4,6,8^ and hematogenous or lymphatic spread of tumor cells.^6,29–31^ The PEG tube is pulled from the upper alimentary tract to the abdominal wall, which may lead to direct contact with tumors located in the oral cavity, oropharynx, or hypopharynx.^8^ Accordingly, we hypothesized that the incidence of esophageal metastasis increases with PEG insertion by similar mechanisms. Placing a PEG using so-called pull methods is widely used in Taiwan, as compared with other techniques. However, there is currently a lack of studies investigating this issue. For the first time, our data confirmed that insertion of a PEG tube in head and neck cancer patients increased the risk of developing subsequent esophageal cancer. *Helicobacter pylori* infection can drive T-cell-mediated immune responses, particularly Th17 inflammation, and result in gastric adenocarcinoma.^32^ There is no previous report of subsequent gastric cancer in a head and neck cancer patient after PEG. The possible mechanism of link between PEG insertion and gastric cancer may be related to immune responses.

The survival and disease control in head and neck cancer patients are affected dramatically by the occurrence of esophageal metastasis.^21,22^ Therefore, prevention of esophageal metastasis is important. Chang et al^8^ hypothesized that pretreatment of PEG tubes using povidone-iodine could diminish the possibility of tumor seeding to the stoma area. Our results showed that hypopharyngeal, oropharyngeal, and laryngeal cancer patients treated with PEG had a higher incidence of subsequent esophageal cancer compared with in the control cohort (Table 2). Particularly, the high HR (3.99, 95% CI = 2.76–4.98) in patients with hypopharyngeal tumors should be noted, and more attention should be paid to such patients. This finding may be related to the anatomic location of these tumors, with the hypopharynx located just above the inlet of the esophagus. On the basis of the findings of the present study, we recommend that surgically placed tubes are more suitable in patients with head and neck cancers, especially in those with cancer of the hypopharynx, oropharynx, and larynx.

This study has a number of strengths. First, the sample size is very large, which enhances the statistical power of the data. We moreover performed stratified analyses according to sex, age, primary tumor location, and comorbidities and assessed a wide range of demographic characteristics. Second, owing to the nationwide database used, which has an extremely high coverage rate, almost all patients had complete follow-up data available. Third, the population-based data used are representative of the general population in Taiwan.

Nevertheless, there are also some limitations in our study. First, this is only a retrospective cohort study, which has a lower statistical quality. Bias from unknown confounders may have affected our results, and a well-designed prospective randomized control study is needed in the future to help establish a causal relationship. Second, the NHIRD does not offer information such as tumor stage and tumor histology, which may have influenced our results. Third, some important clinical information, such as pathology reports, endoscopic findings, and imaging results, was also unavailable because of the patients included in the NHIRD being anonymous. Fourth, secondary esophageal cancer seems to occur in a very small number of head and neck cancer patients (16/1851 cases and 15/3702 controls); although the HR of 2.31 was statistically significant, the clinical relevance at such low incidences should be interpreted with caution. Fifth, patients with esophagitis showed a lower overall difference in the increased risk between the PEG and control groups, and the risk was also lower than that in patients without esophagitis (HR = 2.46); this result may be due to the small number of patients with esophagitis.

In conclusion, head and neck cancer patients with PEG insertion are associated with a higher risk of developing esophageal cancer in Taiwan. Especially, those with hypopharyngeal, oropharyngeal, and laryngeal cancers showed a much higher risk for esophageal cancer. Surgically placed tubes are recommended and physicians should be pay careful attention to this link in such patients.

## References

[1] Lee JH Machtay M Unger LD Prophylactic gastrostomy tubes in patients undergoing intensive irradiation for cancer of the head and neck. *Arch Otolaryngol Head Neck Surg* 1998; 124:871–875. 9708712 10.1001/archotol.124.8.871

[2] Löser C Aschl G Hébuterne X ESPEN guidelines on artificial enteral nutrition: percutaneous endoscopic gastrostomy (PEG). *Clin Nutr* 2005; 24:848–861. 16261664 10.1016/j.clnu.2005.06.013

[3] Ahmed KA Samant S Vieira F Gastrostomy tubes in patients with advanced head and neck cancer. *Laryngoscope* 2005; 115:44–47. 15630364 10.1097/01.mlg.0000150679.60731.bc

[4] Adelson RT Ducic Y Metastatic head and neck carcinoma to a percutaneous endoscopic gastrostomy site. *Head Neck* 2005; 27:339–343. 15712297 10.1002/hed.20159

[5] Ananth S Amin M Implantation of oral squamous cell carcinoma at the site of a percutaneous endoscopic gastrostomy: a case report. *Br J Oral Maxillofac Surg* 2002; 40:125–130. 12180203 10.1054/bjom.2001.0740

[6] Brown MC Cancer metastasis at percutaneous endoscopic gastrostomy stomata is related to the hematogenous or lymphatic spread of circulating tumor cells. *Am J Gastroenterol* 2000; 95:3288–3291. 11095357 10.1111/j.1572-0241.2000.03339.x

[7] Cappell MS Risk factors and risk reduction of malignant seeding of the percutaneous endoscopic gastrostomy track from pharyngoesophageal malignancy: a review of all 44 known reported cases. *Am J Gastroenterol* 2007; 102:1307–1311. 17488255 10.1111/j.1572-0241.2007.01227.x

[8] Chang WK Lin HH Hsieh TY Benefits of the povidone-iodine: simultaneously decrease risk of infection and tumor seeding after percutaneous endoscopic gastrostomy. *Med Hypotheses* 2014; 82:678–680. 24650418 10.1016/j.mehy.2014.03.002

[9] Ellrichmann M Sergeev P Bethge J Prospective evaluation of malignant cell seeding after percutaneous endoscopic gastrostomy in patients with oropharyngeal/esophageal cancers. *Endoscopy* 2013; 45:526–531. 23780843 10.1055/s-0033-1344023

[10] Huang AT Georgolios A Espino S Percutaneous endoscopic gastrostomy site metastasis from head and neck squamous cell carcinoma: case series and literature review. *J Otolaryngol Head Neck Surg* 2013; 42:20. 23672761 10.1186/1916-0216-42-20 PMC3651229

[11] Sousa AL Sousa D Velasco F Rare complication of percutaneous endoscopic gastrostomy: Ostomy metastasis of esophageal carcinoma. *World J Gastrointest Oncol* 2013; 5:204–206. 24244807 10.4251/wjgo.v5.i11.204 PMC3828636

[12] Tsai JK Schattner M Percutaneous endoscopic gastrostomy site metastasis. *Gastrointest Endosc Clin N Am* 2007; 17:777–786. 17967381 10.1016/j.giec.2007.07.012

[13] Hussain A Woolfrey S Massey J Percutaneous endoscopic gastrostomy. *Postgrad Med J* 1996; 72:581–585. 8977937 10.1136/pgmj.72.852.581 PMC2398609

[14] Schwartz LH Ozsahin M Zhang GN Synchronous and metachronous head and neck carcinomas. *Cancer* 1994; 74:1933–1938. 8082099 10.1002/1097-0142(19941001)74:7<1933::aid-cncr2820740718>3.0.co;2-x

[15] Muto M Hironaka S Nakane M Association of multiple Lugol-voiding lesions with synchronous and metachronous esophageal squamous cell carcinoma in patients with head and neck cancer. *Gastrointest Endosc* 2002; 56:517–521. 12297767 10.1067/mge.2002.128104

[16] Goldstein HM Zornoza J Association of squamous cell carcinoma of the head and neck with cancer of the esophagus. *AJR Am J Roentgenol* 1978; 131:791–794. 101029 10.2214/ajr.131.5.791

[17] Abemayor E Moore DM Hanson DG Identification of synchronous esophageal tumors in patients with head and neck cancer. *J Surg Oncol* 1988; 38:94–96. 3288813 10.1002/jso.2930380207

[18] Ina H Shibuya H Ohashi I The frequency of a concomitant early esophageal cancer in male patients with oral and oropharyngeal cancer. Screening results using Lugol dye endoscopy. *Cancer* 1994; 73:2038–2041. 7512440 10.1002/1097-0142(19940415)73:8<2038::aid-cncr2820730804>3.0.co;2-x

[19] Cahan WG Castro EB Rosen PP Separate primary carcinomas of the esophagus and head and neck region in the same patient. *Cancer* 1976; 37:85–89. 1247970 10.1002/1097-0142(197601)37:1<85::aid-cncr2820370112>3.0.co;2-j

[20] Shibuya H Hisamitsu S Shioiri S Multiple primary cancer risk in patients with squamous cell carcinoma of the oral cavity. *Cancer* 1987; 60:3083–3086. 3677030 10.1002/1097-0142(19871215)60:12<3083::aid-cncr2820601237>3.0.co;2-l

[21] McDonald S Haie C Rubin P Second malignant tumors in patients with laryngeal carcinoma: diagnosis, treatment, and prevention. *Int J Radiat Oncol Biol Phys* 1989; 17:457–465. 2674074 10.1016/0360-3016(89)90095-3

[22] Cooper JS Pajak TF Rubin P Second malignancies in patients who have head and neck cancer: incidence, effect on survival and implications based on the RTOG experience. *Int J Radiat Oncol Biol Phys* 1989; 17:449–456. 2674073 10.1016/0360-3016(89)90094-1

[23] Chiang TL Taiwan's 1995 health care reform. *Health Policy* 1997; 39:225–339. 10165463 10.1016/s0168-8510(96)00877-9

[24] Chen YC Wu JC Chen TJ Reduced access to database. A publicly available database accelerates academic production. *BMJ* 2011; 342:d637. 21285219 10.1136/bmj.d637

[25] Huang WY Lin CC Jen YM Association between adult otitis media and nasopharyngeal cancer: a nationwide population-based cohort study. *Radiother Oncol* 2012; 104:338–342. 22981611 10.1016/j.radonc.2012.08.015

[26] Lin KT Huang WY Lin CC Subsequent risk of nasopharyngeal carcinoma among patients with allergic rhinitis: a nationwide population-based cohort study. *Head Neck* 2015; 37:413–417. 24435940 10.1002/hed.23617

[27] Liu FC Huang WY Lin TY Inverse association of parkinson disease with systemic lupus erythematosus: a nationwide population-based study. *Medicine* 2015; 94:e2097. 26579824 10.1097/MD.0000000000002097 PMC4652833

[28] Meurer MF Kenady DE Metastatic head and neck carcinoma in a percutaneous gastrostomy site. *Head Neck* 1993; 15:70–73. 8416862 10.1002/hed.2880150116

[29] Saito T Iizuka T Kato H Esophageal carcinoma metastatic to the stomach. A clinicopathologic study of 35 cases. *Cancer* 1985; 56:2235–2241. 4052968 10.1002/1097-0142(19851101)56:9<2235::aid-cncr2820560917>3.0.co;2-0

[30] Strodel WE Kenady DE Zweng TN Avoiding stoma seeding in head and neck cancer patients. *Surg Endosc* 1995; 9:1142–1143. 8553223 10.1007/BF00189008

[31] Bureau of Health Promotion, Department of Health. Cancer Registry Annual Report; Taiwan 2009 Available at: http://tcr.cph.ntu.edu.tw/uploadimages/Y98-ALL.pdf [accessed November 19, 2015]. 2012; p. 30 [Chinese].

[32] Amedeo A Fabio M Chiara DB Helicobacter pylori secreted peptidyl prolyl cis, trans-isomerase drives Th17 inflammation in gastric adenocarcinoma. *Intern Emerg Med* 2014; 9:303–309. 23054412 10.1007/s11739-012-0867-9

